# Leptomeningeal Metastasis in Non-Small-Cell Lung Cancer with Actionable Genomic Alterations: Pathogenesis, Diagnosis, and Emerging Therapeutic Strategies

**DOI:** 10.3390/cancers18132169

**Published:** 2026-07-06

**Authors:** Sung-Won Lim, Bo Mi Ku, Myung-Ju Ahn

**Affiliations:** Division of Hematology and Oncology, Department of Medicine, Hanyang University Medical Center, Hanyang University College of Medicine, Seoul 04763, Republic of Koreabomiku@hanyang.ac.kr (B.M.K.)

**Keywords:** leptomeningeal metastasis, meningeal carcinomatosis, non-small-cell lung cancer, molecular targeted therapy

## Abstract

Leptomeningeal metastasis (LM) is a severe and increasingly recognized complication of advanced non-small-cell lung cancer (NSCLC), particularly in patients with actionable genomic alterations. Although LM has historically been associated with poor outcomes, molecularly targeted systemic therapies with improved central nervous system (CNS) activity are reshaping its therapeutic landscape. This review summarizes current concepts in the pathogenesis, diagnosis, and risk stratification of LM, focusing on systemic treatment strategies for NSCLC harboring actionable driver alterations. We highlight the rationale and emerging evidence for next-generation tyrosine kinase inhibitors targeting EGFR, ALK, and other oncogenic drivers, and discuss their role as the cornerstone of LM management. Intrathecal chemotherapy, immunotherapy, and radiotherapy are also reviewed within a risk-adapted treatment framework. An individualized approach integrating molecular profiling, neurological status, and disease distribution is essential to optimize outcomes. Prospective studies are needed to refine systemic treatment strategies and establish evidence-based algorithms for this high-risk population.

## Simple Summary

Leptomeningeal metastasis is an increasingly important complication of advanced NSCLC, especially in tumors with actionable genomic alterations. This review summarizes current diagnostic frameworks, risk stratification, and treatment strategies, with emphasis on CNS-penetrant targeted therapies. We also discuss TKI resistance, CSF-based molecular profiling and emerging treatments beyond TKIs.

## 1. Introduction

Leptomeningeal metastasis (LM) is defined by dissemination of tumor cells within the leptomeninges and subarachnoid space. The lifetime risk of LM in metastatic solid tumors approaches 10% [1,2] and its detection is increasing because of prolonged survival with modern systemic therapies and improved neuroimaging [3]. Most cases arise from breast cancer, lung cancer, or melanoma [4,5]. This review provides an overview of LM, with emphasis on therapeutic strategies for NSCLC harboring actionable genomic alterations.

## 2. Pathogenesis

### 2.1. Anatomical Process

The meninges consist of the dura mater, arachnoid, and pia mater [6]. The leptomeninges comprise the arachnoid and pia mater, and the cerebrospinal fluid (CSF) circulates within the subarachnoid space between them. CSF is produced primarily by the choroid plexus, a specialized epithelial structure with tight junctions surrounding fenestrated capillaries [7].

**Figure 1:**
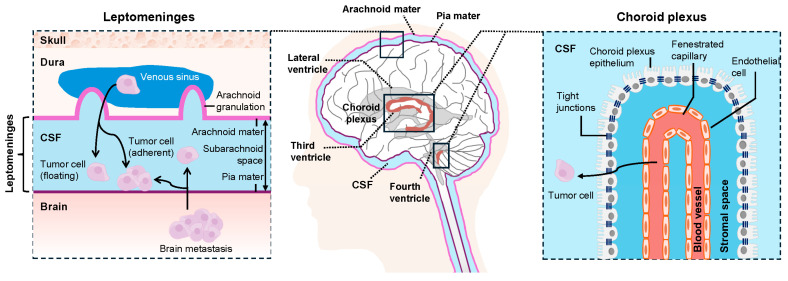
Simplified schematic of LM and route of LM involvement. The leptomeninges consists of the pia mater, subarachnoid space, and arachnoid mater. Cerebrospinal fluid is produced by choroid plexus located within the ventricles and circulated from the ventricles through the subarachnoid spaces of the brain and spinal cord until it is reabsorbed into the venous system. The possible anatomical routes of cancer spread to the leptomeninges: (1) Direct migration of tumor cells from adjacent brain parenchymal metastasis. (2) Hematogenous spread often by way of venous vasculature. (3) Entry into the CSF through the fenestrated capillaries of the choroid plexus (blood–CSF barrier). Following entry into the CSF, tumor cells may disseminate through CSF flow, either remaining as free-floating cells within the CSF or adhering to the pia mater of the brain and spinal cord.

Leptomeningeal involvement can arise through direct extension from adjacent brain parenchymal metastases, hematogenous dissemination via the venous circulation, or tumor-cell entry through the fenestrated vasculature of the choroid plexus. These anatomical routes are facilitated by metastatic programs such as epithelial–mesenchymal transition, which promotes loss of cell adhesion and polarity, enhances tumor-cell migration and invasion, and enables circulating tumor cells to seed distant organs. After gaining access to the central nervous system, tumor cells may cross the blood–brain or blood–CSF barriers and colonize the subarachnoid space, where subsequent adaptation to the CSF microenvironment supports LM progression [8,9]. A simplified schematic of LM development is shown in Figure 1.

### 2.2. Cancer Specific Process

Metastatic dissemination is non-random and reflects tumor-specific organotropism [10]. Breast cancer, lung cancer, and melanoma account for approximately 80% of solid tumors associated with LM [11]. In breast cancer, lobular histology and triple-negative disease confer higher LM risk. In NSCLC, particularly adenocarcinoma, actionable alterations such as EGFR mutations, ALK and ROS1 rearrangements, MET exon 14 skipping, RET fusions, and NTRK fusions are associated with an increased risk of CNS and leptomeningeal dissemination. Reported cumulative LM prevalence is 11.1% in EGFR-mutant, 11.2% in ALK-rearranged, 16.0% in ROS1-rearranged, and 12.3% in ERBB2-altered NSCLC, compared with 3.6% in molecularly unselected disease [12]. In MET exon 14-altered NSCLC, CNS metastases occurred in 36% of patients in one series, with LM in 17% [13]. Data for RET- and NTRK fusion-positive NSCLC remain limited, although baseline CNS involvement is frequently reported [14].

The genetic features that distinguish LM from solid brain metastases remain incompletely defined. Whole-exome sequencing of eight NSCLC LM samples identified EGFR mutations in six patients (75%) and TP53 mutations in four (50%), compared with 26 solid brain metastases [15]. A meta-analysis of LM across NSCLC, breast cancer, and melanoma identified recurrent alterations in TP53, PTEN, PIK3CA, IL7R, and KMT2D [16].

## 3. Diagnosis

The EANO-ESMO guidelines proposed a diagnostic classification for LM based on neurological findings, neuroimaging, and CSF analysis to support clinical decision-making [17].

### 3.1. Neurological Examination

Clinical manifestations vary according to the involved CNS compartment. Multifocal neurological deficits are highly suggestive, but symptoms may be isolated or subtle, and neurological examination may occasionally be normal. Typical manifestations include headache, nausea, vomiting, cognitive or mental-status changes, gait disturbance, cranial nerve palsies with diplopia or visual/auditory impairment, radicular pain or weakness, urinary dysfunction, cauda equina syndrome, and focal or radiating neck or back pain.

### 3.2. Radiographic Findings

Contrast-enhanced cerebrospinal MRI is the gold-standard imaging modality for diagnosis and follow-up of suspected or confirmed LM. MRI patterns include linear leptomeningeal enhancement, nodular enhancement, mixed linear and nodular disease, isolated hydrocephalus, and absence of radiological evidence of LM. Because lesions are often small and geometrically complex, quantitative measurement is frequently impractical. LM may be classified as measurable when at least one nodular lesion is ≥5 × 5 mm in orthogonal diameters on two planes; all other MRI abnormalities are considered non-measurable. CT should be reserved for MRI contraindications or emergencies, such as suspected CSF flow obstruction or intracranial hemorrhage. FDG PET-CT is not recommended for LM diagnosis because of technical limitations.

### 3.3. CSF Analysis

Lumbar puncture should be performed after neuroimaging to reduce the risk of herniation or procedure-related complications. Diagnostic yield is improved by collecting an adequate CSF volume (preferably >10 mL, and at least 5 mL), processing the sample within 30 min, and avoiding hemorrhagic contamination. If initial cytology is negative, a second lumbar puncture under optimized conditions is recommended; sensitivity increases from approximately 50–60% after the first sample to about 80% after repeat sampling [18]. CSF cytological findings should be categorized as positive, indicating the presence of malignant cells; equivocal, referring to the identification of “suspicious” or “atypical” cells; or negative, indicating the absence of malignant cells. Although malignant cells in the CSF remain the most definitive method for confirming LM, cytology has important limitations. Tumor burden is not uniformly distributed, and results may vary by sampling site. In 60 patients with simultaneous ventricular and lumbar CSF sampling, paired cytology was discordant in 32%, with false-negative rates of 17% for lumbar CSF and 20% for ventricular CSF [19]. Predominant cisternal or cranial disease may therefore yield false-negative lumbar cytology. Negative CSF cytology should be interpreted in the clinical and radiographic context.

Liquid biopsy may provide useful adjunctive information for diagnosis and monitoring. CSF circulating tumor cells (CTCs) and circulating tumor DNA (ctDNA) are the most developed biomarkers. Genomic alterations can be detected using DNA-based microarrays [20], digital or real-time PCR, targeted amplicon sequencing, whole-exome sequencing, or next-generation sequencing [21,22,23]. In a prospective cohort of patients with EGFR-mutant NSCLC undergoing CSF analysis for suspected LM, droplet digital PCR showed higher sensitivity than real-time PCR (94.3% vs. 75.6%) [24]. CSF-CTC detection using the CellSearch immunomagnetic platform has also shown greater sensitivity than conventional cytology and MRI in several studies [25,26,27], with one assay reporting 88.9% sensitivity and 100% specificity [28].

## 4. Staging and Risk Assessment

According to EANO-ESMO criteria, LM is first classified by cytological or histological verification: type I when malignant cells are confirmed and type II when findings are equivocal or negative. Integrating clinical and radiographic features further stratifies LM as confirmed, probable, or possible. Le Rhun et al. showed that survival differed by diagnostic certainty, with possible LM showing the longest survival (5.1 months; IQR, 2.4–14.2) and confirmed LM the shortest (2.3 months; IQR, 0.8–4.9). Type II LM was also associated with better survival than type I disease [29].

The NCCN stratifies patients into poor-risk and good-risk groups [30]. Poor-risk features include Karnofsky Performance Status (KPS) < 60, multiple serious neurological deficits, extensive systemic disease with few treatment options, bulky CNS disease, or encephalopathy related to neoplastic meningitis. Good-risk patients have KPS ≥ 60, no major neurological deficits, minimal systemic disease, and reasonable systemic treatment options. In cytology-positive LM, median survival was 15.5 weeks in patients with good performance status versus 6 weeks in those with poor performance status [31].

## 5. Treatments

For good-risk patients, systemic therapy, intra-CSF therapy, and/or radiotherapy may be considered. Poor-risk patients are generally managed with palliative or best supportive care. However, standardized treatment remains difficult because prospective data are limited. Historically, outcomes in NSCLC-associated LM were poor, partly because many systemic therapies have limited penetration across the blood–brain barrier (BBB) [32].

The development of later-generation TKIs has changed this therapeutic framework. Because EGFR-mutant and ALK-rearranged NSCLC have a high propensity for LM and respond to targeted therapies, CNS-penetrant systemic agents may be appropriate even in selected poor-risk patients when actionable drivers are present. Intra-CSF therapy and radiotherapy remain options when clinically justified.

This review focuses on TKI-based treatment for LM in NSCLC with actionable genomic alterations, including resistance mechanisms and emerging systemic strategies. General treatment modalities, such as intrathecal chemotherapy and radiotherapy, are also briefly reviewed.

- Molecularly Targeted TKIs for NSCLC With Actionable Genomic Alterations

**Figure 2:**
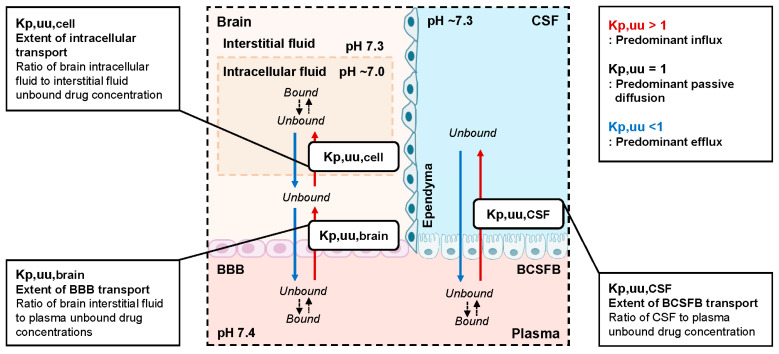
Conceptual overview of BBB and intra brain drug transport and distribution. BBB, blood–brain barrier. BCSFB, blood cerebrospinal fluid barrier. CSF, cerebrospinal fluid.

Preclinical assessment of CNS drug exposure is an important step in estimating BBB permeability. The unbound partition coefficient (K_p,uu_) is widely used to quantify CNS drug distribution [33]. K_p,uu,brain_ is the steady-state ratio of unbound drug concentration in brain parenchyma to that in plasma [34]; whereas, K_p,uu,CSF_ is the corresponding ratio in CSF. Figure 2 provides a conceptual overview, and available K_p,uu,brain_ and K_p,uu,CSF_ data for CNS-penetrant TKIs are summarized in Table 1 [35].

In NSCLC with actionable genomic alterations and LM, BBB and blood–CSF barrier penetration are important determinants of TKI activity, but CSF drug concentration alone does not predict response. Early generation EGFR and ALK inhibitors, including gefitinib and crizotinib, achieve low CSF exposure and are associated with CNS or LM progression. Dose-intensified approaches, such as pulsatile high-dose erlotinib, were developed to increase CSF exposure. Newer-generation TKIs, including osimertinib, lazertinib, lorlatinib, and tepotinib, show improved CNS or CSF activity. Nevertheless, efficacy is also influenced by CSF-specific driver or resistance alterations, unbound active drug fraction, efflux transporter activity, tumor burden, CSF flow, and compartmental heterogeneity. Pharmacokinetic data should therefore be interpreted with CSF genotyping and integrated clinical-radiographic assessment.

- EGFR-Mutant NSCLC

Patients with oncogenic driver alterations, particularly EGFR-mutant tumors, have a higher propensity for LM [36]. Osimertinib is a third-generation EGFR TKI active against classical EGFR-activating mutations and the T790M resistance mutation, and it is the standard first-line therapy for metastatic EGFR-mutant NSCLC. Compared with first- and second-generation TKIs, osimertinib has superior efficacy in patients with asymptomatic or stable CNS metastases [37,38]. This clinical benefit is supported by preclinical data showing greater exposure in the brain parenchyma. By contrast, CSF concentrations of osimertinib may be comparable to those of erlotinib [35]. Pulsatile erlotinib strategies were developed to overcome limited CSF penetration and have produced response rates of approximately 30–60% [39,40,41]. High-dose osimertinib (160 mg/day) also showed activity in the BLOOM study, with a 62% LM response rate in heavily pretreated patients [42], and additional studies have reported activity with this dose [43]. Favorable outcomes have also been reported with standard-dose osimertinib 80 mg/day [44,45]. Overall, standard-dose osimertinib remains the preferred systemic option for EGFR-mutant NSCLC with LM; whereas, dose-escalated osimertinib or pulsatile erlotinib may be considered in selected patients. EGFR-TKI studies are summarized in Table 2.

- ALK-Rearranged NSCLC

ALK rearrangement is a clinically important oncogenic alteration in NSCLC. ALK-rearranged disease is treated with ALK TKIs, and newer-generation agents generally have improved CNS penetration [46,47,48]. Crizotinib, the first approved ALK inhibitor, has poor CNS penetration [49]. In the ALEX phase III trial, alectinib improved outcomes compared with crizotinib in patients with baseline CNS disease, including asymptomatic brain metastases or LM [47]. Lorlatinib, a third-generation ALK/ROS1 inhibitor, retains activity against a broad spectrum of crizotinib-associated resistance mutations and has robust BBB penetration in preclinical and clinical studies [50,51,52]. Several case reports have described marked responses to lorlatinib in previously treated ALK-positive NSCLC with LM [53,54].

- MET Exon 14-Skipping NSCLC

MET exon 14 (METex14) skipping is an actionable driver in NSCLC. Its frequency is approximately 2.4% in adenocarcinoma or nonsquamous NSCLC, 12.0% in sarcomatoid carcinoma, and 1.3% in squamous histology. At diagnosis, 17% of patients with METex14-altered lung cancer have CNS metastases, and 19% develop CNS metastases during the disease course [13]. Crizotinib has shown limited intracranial activity in small reports, including cases with LM, but the evidence remains weak. Selective type Ib MET TKIs, including capmatinib and tepotinib, are approved MET-directed therapies. In GEOMETRY, capmatinib showed clinically meaningful intracranial activity in patients with brain metastases [55]. In VISION, tepotinib produced robust systemic and intracranial outcomes [56].

Whether patients with LM were included in these trials is unclear. Case reports have described symptomatic improvement of LM with tepotinib, including one study measuring plasma and CSF tepotinib concentrations and supporting CNS penetration [57,58]. NCCN guidelines recommend tepotinib for MET exon 14-skipping NSCLC [30].

- ROS1-, BRAF-, NTRK-, and RET-Altered NSCLC

ROS1 rearrangements, BRAF mutations, NTRK fusions, and RET fusions are actionable NSCLC alterations with targeted therapies that have demonstrated activity against CNS metastases. However, evidence in LM remains limited to small case series and case reports. More robust clinical data are needed to define their role in this setting.

- Therapeutic Resistance to TKIs in Oncogene-Driven NSCLC with LM

Despite clinically meaningful survival benefits from TKIs in oncogene-driven NSCLC with LM, acquired resistance eventually develops in most patients. Several studies have investigated resistance by comparing CSF and plasma genotypes in patients with new or progressive LM during or after targeted therapy.

van der Wel et al. analyzed paired CSF and plasma samples from patients with EGFR-mutant NSCLC who developed new or progressive LM during osimertinib therapy [59]. Driver mutations were detected in CSF in 93% of patients, but osimertinib resistance mechanisms were identified in only 27%, and no targetable resistance mechanisms were observed. Zheng et al. evaluated CSF and plasma ctDNA in 81 patients with LM progression on osimertinib and found that CSF-private alterations were substantially more frequent than plasma-private alterations [60], suggesting CSF-specific genomic evolution. Xie et al. analyzed 116 patients with EGFR-mutant NSCLC and LM after resistance to third-generation EGFR TKIs [61]. In paired samples, alterations were more frequently detected in CSF than in plasma (97.9% vs. 60.4%), and ctDNA abundance was higher in CSF (0.76 vs. 0.21; *p* < 0.001). Common third-generation EGFR-TKI resistance mutations were not detected in either compartment. CSF-enriched genes were involved in cell-cycle (80% vs. 23%; *p* < 0.001), KRAS/RAF (27% vs. 8%; *p* < 0.001), and WNT pathways (18% vs. 4%; *p* = 0.002).

Together, these studies indicate that systemic resistance mechanisms after third-generation EGFR-TKI therapy may not fully explain LM progression. Even CSF ctDNA identifies only a subset of resistance events, suggesting that many cases involve unknown, non-targetable, or non-genomic mechanisms. Nonetheless, genomic alterations are more readily detected in CSF than in plasma, and CSF ctDNA appears more sensitive for detecting somatic alterations and metastasis-related pathways. Plasma ctDNA alone may therefore be insufficient at LM progression, and CSF-based profiling should be considered when feasible.

The CSF tumor microenvironment may also contribute to EGFR-TKI resistance and LM progression. Li et al. performed single-cell RNA sequencing of CSF from patients with EGFR-mutant NSCLC and CNS metastases, including LM [62]. In addition to tumor-cell DNA alterations, high CD47 expression and activation of the STAT3/CD47-SIRPA axis may mediate off-target EGFR-TKI resistance through interactions with tumor-associated macrophages. In LM, the authors observed increased CSF monocytes and macrophages with M2-like polarization, along with immunosuppressive and exhausted T cell phenotypes. These findings suggest that LM progression and osimertinib resistance may be driven by both genomic alterations and CSF microenvironmental factors, including lipid-associated macrophages, macrophage-tumor interactions, and CD47-SIRPA signaling.

The optimal strategy after third-generation EGFR-TKI progression in EGFR-mutant NSCLC with LM is not established. Limited data suggest that high-dose EGFR-TKI therapy, alone or with antiangiogenic therapy, intrathecal chemotherapy, or whole-brain radiotherapy, may provide benefit. In one real-world study, high-dose EGFR-TKI therapy with or without additional modalities improved survival compared with standard-dose EGFR-TKI-based combinations after third-generation EGFR-TKI failure [63]. Another study showed that high-dose furmonertinib plus bevacizumab improved median intracranial progression-free survival (6.77 vs. 4.04 months) and overall survival (15.31 vs. 7.10 months) compared with high-dose furmonertinib alone [64].

Amivantamab, an EGFR-MET bispecific antibody, is an emerging strategy in EGFR-mutant NSCLC. In the phase III MARIPOSA trial, first-line amivantamab plus lazertinib significantly prolonged progression-free survival compared with osimertinib in advanced NSCLC harboring EGFR exon 19 deletion or L858R mutations [65]. Given its CNS activity, this regimen may also have potential in LM [66,67,68]. In a phase II study of 21 heavily pretreated patients with EGFR-mutant NSCLC and LM, including patients previously treated with osimertinib, lazertinib plus amivantamab achieved a response rate of approximately 30% [69].

- Beyond TKIs: Emerging Antibody-Based Therapeutics in NSCLC With Actionable Genomic Alterations

HER3 promotes tumor growth, proliferation, invasion, metastasis, and chemotherapy resistance. HER3 expression has been reported in lung cancer, breast cancer, melanoma, and other tumors [70], and HER3 overexpression is frequent in CNS metastases, including brain metastases from breast cancer and NSCLC [71]. Patritumab deruxtecan (HER3-DXd) is an intravenous HER3-directed antibody-drug conjugate composed of a fully human anti-HER3 IgG1 monoclonal antibody linked to a topoisomerase I inhibitor payload via a tetrapeptide-based cleavable linker. In a prospective phase II study of 20 patients with treatment-naive or post-radiotherapy progressive LM from solid tumors, the most common primary cancers were breast cancer (60%) and lung cancer (30%). HER3-DXd achieved a 3-month overall survival rate of 65%, Kaplan–Meier-estimated 3- and 6-month overall survival rates of 69.6% and 58.9%, and an intracranial clinical benefit rate of 50%, suggesting clinically meaningful activity in LM.

Trastuzumab deruxtecan (T-DXd; formerly DS-8201a) has demonstrated substantial efficacy in HER2-positive solid tumors, but its activity in LM requires further validation. The phase II DEBBRAH trial demonstrated clinically meaningful CNS activity in patients with HER2-positive or HER2-low advanced breast cancer with brain metastases or leptomeningeal carcinomatosis [72]. Although these data are not from NSCLC, they support the broader concept that selected ADCs can exert activity within the CNS and CSF compartments. Further studies are needed to determine whether HER3-DXd, T-DXd, or other ADCs provide meaningful benefit in NSCLC-associated LM (Table 3).

- Immunotherapy

The activity of immune checkpoint inhibitors (ICIs) in brain metastases from melanoma, lung cancer, and breast cancer has prompted interest in their use for LM [73,74,75]. ICI strategies include monotherapy, dual checkpoint blockade, and combinations with chemotherapy or radiotherapy. Most NSCLC ICI trials allowed patients with controlled, asymptomatic brain metastases but excluded LM. Evidence for systemic ICIs in LM is therefore limited. Retrospective series and case reports suggest occasional prolonged survival, but no consistent clinical benefit [76,77]. In a phase II trial of pembrolizumab for LM from solid tumors, including three NSCLC cases, the CNS response rate was 38% [78]. Another phase II trial of ipilimumab plus nivolumab in 18 patients with solid-tumor LM met its 3-month survival endpoint in eight patients, although one-third developed grade 3 or 4 adverse events [79].

Overall, LM responses to intravenous ICIs are heterogeneous and often modest. This variability may reflect immune-evasion programs amplified within the CSF compartment. Tumor cells in the leptomeninges encounter a distinct niche characterized by limited nutrients, altered oxygen tension, dynamic oxidative stress, metabolic adaptation, and immunosuppression. During metastatic dissemination, tumor cells are exposed to increased and dynamically fluctuating oxidative stress in foreign microenvironments. Disseminated tumor cells encounter increased oxidative stress in foreign microenvironments, and reactive oxygen species (ROS) levels fluctuate dynamically throughout the metastatic cascade. This redox stress imposes context-dependent selective pressure, allowing surviving tumor cells to sustain metastatic potential through metabolic and epigenetic reprogramming [80]. ROS also contribute to immune evasion by promoting the expansion and suppressive function of myeloid-derived suppressor cells, downregulating surface antigens such as MHC class I, and remodeling the tumor microenvironment in ways that impair cytotoxic immune surveillance. In parallel, nutrient and oxygen deprivation, oncometabolite accumulation, and a hostile stromal–redox milieu restrict effector T cell metabolism and promote CD8-positive T cell exhaustion and functional depletion, key features of immune-cold tumors [81]. Consistent with this, single-cell analyses in NSCLC have shown that, compared with brain parenchymal metastases, LM exhibits an immune-cold phenotype marked by depletion of CD8-positive effector T cells, plasma cells, dendritic cells, and pro-inflammatory macrophages [82,83].

These limitations have stimulated interest in engineered cellular and vaccine-based therapies. gamma-delta T cells may be advantageous in rapidly progressive CNS disease because of MHC-independent tumor recognition, cytotoxicity, and off-the-shelf feasibility. QH104 is an allogeneic B7-H3-targeted CAR Vgamma9Vdelta2 T cell product with in vitro activity against B7-H3-positive tumor cells. In a phase I study of intrathecal QH104 for LM, Ma et al. treated three patients, including two with EGFR L858R-mutant lung adenocarcinoma [84]. B7-H3 CAR gamma-delta T cells persisted in CSF for at least one week, increased IFN-gamma levels, and were associated with CSF immune remodeling, including expansion of inflammatory macrophages followed by lymphocyte recruitment. CAR-macrophage therapy is also conceptually attractive for LM. In a first-in-human phase I trial, the autologous anti-HER2 CAR-macrophage product CT-0508 showed biologic activity in HER2-overexpressing advanced solid tumors through phagocytosis, cytokine release, antigen presentation, and TME activation [85]. Serial biopsies showed CAR-M trafficking and immune remodeling with CD8-positive T cell expansion. In a preclinical breast cancer LM model, intrathecal HER2/HER3 dendritic cell vaccination increased CSF CD4- and CD8-positive T cell infiltration and induced IFN-gamma and IL-18 responses [86]. Although these approaches remain investigational and are not yet established in NSCLC-associated LM, they support further study of cellular and vaccine-based therapies to overcome the immunosuppressive CSF microenvironment.

- Intrathecal chemotherapy

A pooled analysis suggested that intrathecal chemotherapy can be effective in NSCLC-associated LM [87], but the optimal agent, dose, and schedule remain undefined. Agents used include temozolomide, thiotepa, topotecan, etoposide, cytarabine, and methotrexate [88,89,90,91,92,93]. Treatment may be delivered by lumbar puncture or intraventricular injection through an Ommaya reservoir. Because intra-CSF therapy can cause substantial toxicity and procedure-related complications, it is generally reserved for patients with good performance status.

A phase I/II trial evaluated intrathecal pemetrexed with dexamethasone in EGFR-mutant NSCLC with TKI-progressive LM [94]. The recommended intrathecal pemetrexed dose was 50 mg. In phase II, the clinical response rate was 84.6%. Higher doses were associated with myelosuppression; whereas, nausea, vomiting, and neurotoxicity occurred at the recommended dose. Several ongoing trials are also evaluating intrathecal immunotherapy for leptomeningeal disease.

- Radiotherapy (RT)

Radiotherapy is used for symptom palliation, correction of CSF flow obstruction, and debulking before chemotherapy [95]. Involved-field radiotherapy (IFRT), including whole-brain radiotherapy (WBRT) or focal spine radiotherapy, may be used for focal disease control or neurological symptom palliation. Craniospinal irradiation (CSI) can be considered in selected patients with good performance status and controlled extracranial disease.

IFRT can improve symptoms but has not consistently improved overall survival, and out-of-field progression is common [96]. In a retrospective study of 51 patients with EGFR-mutant NSCLC and LM, WBRT did not improve objective response rate or disease control rate [97]. By contrast, another retrospective cohort of 149 NSCLC patients with LM suggested improved prognosis with WBRT [98]. Overall, WBRT data are inconsistent; benefit is most likely limited to symptom palliation and selected good-risk patients rather than poor-risk patients [99,100,101].

Focal spine radiotherapy may be directed to symptomatic sites or regions of CSF flow obstruction, particularly in patients with radiculopathy, cauda equina syndrome, or other focal neurological deficits [102].

CSI treats the entire craniospinal CSF space but is associated with substantial toxicity, particularly myelosuppression; thus, it is restricted to carefully selected patients. Proton CSI may reduce dose to normal organs and vertebral bodies [103]. In a randomized phase II trial of 63 patients with LM from NSCLC or breast cancer, proton CSI improved CNS progression-free survival and overall survival compared with IFRT [104]. In selected NCCN good-risk patients, proton-based CSI may be considered when comprehensive CNS and CSF disease control is desired.

### Response Assessment Criteria

Response assessment should integrate neurological examination, contrast-enhanced cerebrospinal imaging, and CSF cytology. Because LM lesions are often difficult to measure, treatment response should not rely on radiographic measurements alone.

Under EANO-ESMO criteria, response requires improvement in all three domains; whereas, deterioration in any domain should raise concern for progression; unequivocal radiographic worsening is considered definitive progression [17]. This framework supports pragmatic treatment decisions in routine practice.

**Figure 3:**
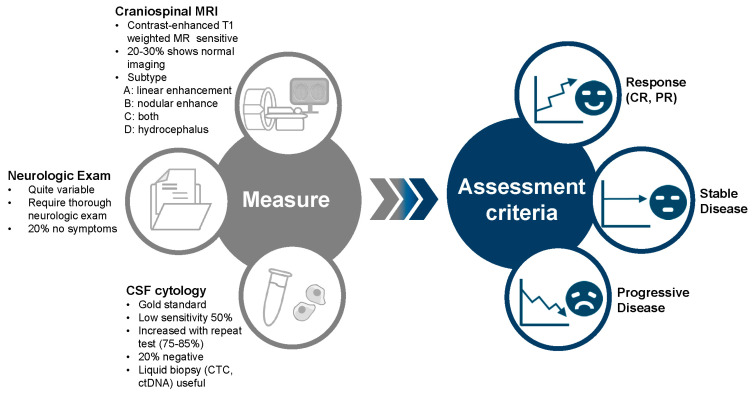
Summarized response assessment of EANO ESMO guidelines for leptomeningeal metastatic disease and RANO LM Clinical, radiological imaging, and CSF cytology evaluations should be carried out at baseline and at defined timepoints hereafter to assess response. EANO ESMO, European Association of Neuro Oncology European Society of Medical Oncology; RANO LM, Response assessment in Neuro Oncology leptomeningeal metastasis; CTC, circulating tumor cell; ctDNA, circulating tumor DNA.

The RANO working group similarly recommends standardized neurological examination, CSF cytology or flow cytometry, and radiographic assessment [105]. RANO-LM uses structured neurological scoring and categorized MRI findings, which are well suited for clinical trials but may be difficult to apply systematically in real-world LM care. The EANO-ESMO and RANO-LM response frameworks are summarized in Figure 3.

These limitations highlight the need for objective, quantifiable response biomarkers and for multidisciplinary interpretation of clinical, radiographic, and CSF findings.

## 6. Discussion

LM is an increasingly recognized complication of advanced NSCLC and is disproportionately enriched in tumors with actionable genomic alterations. Although cumulative LM prevalence is approximately 3–4% in molecularly unselected NSCLC, it is higher in EGFR-mutant, ALK-rearranged, and ROS1-rearranged tumors; clinically relevant CNS involvement is also reported in MET exon 14-altered, RET fusion-positive, and NTRK fusion-positive NSCLC. With prolonged survival from effective targeted therapies, oncogene-driven LM has become a growing clinical challenge. However, evidence remains limited because patients with LM, particularly symptomatic CNS disease, are often excluded from pivotal trials. Current decisions therefore rely largely on retrospective series, single-arm studies, subgroup analyses, and case reports.

Later-generation CNS-penetrant TKIs are central to treatment for oncogene-driven LM. Osimertinib in EGFR-mutant NSCLC, lorlatinib in ALK-rearranged disease, and tepotinib in MET exon 14-altered disease have shown meaningful intracranial or leptomeningeal activity, and dose-intensified approaches may benefit selected patients. However, CSF drug concentration alone does not reliably predict LM efficacy because response also depends on efflux transporter activity, unbound active drug fraction, CSF flow, tumor burden, and compartment-specific genomic heterogeneity. Pharmacokinetic data should be interpreted with CSF genotyping and integrated clinical-radiographic assessment.

The leptomeningeal compartment may evolve semi-independently from systemic disease. Paired CSF and plasma analyses show more frequent and higher-abundance genomic alterations in CSF ctDNA, with many CSF-private events. Yet canonical TKI-resistance mechanisms are detected in only a subset of patients, implying that non-targetable, unknown, or microenvironment-mediated processes also contribute to LM progression. These include M2-polarized macrophages, exhausted T cell phenotypes, and CD47-SIRPA signaling within an immunosuppressive CSF niche. These findings support CSF-based molecular profiling in clinical decision-making and trial design whenever feasible.

Therapies beyond TKIs are promising but immature. Amivantamab plus lazertinib has shown encouraging activity in heavily pretreated EGFR-mutant LM, and ADCs such as HER3-DXd and T-DXd support the concept that selected antibody-based therapies may act within CNS and CSF compartments, although much supporting evidence derives from breast cancer. ICIs show heterogeneous and generally modest activity, consistent with an immune-cold, lymphocyte-depleted leptomeningeal microenvironment. Engineered cellular and vaccine-based approaches, including B7-H3-targeted CAR gamma-delta T cells, CAR-macrophages, and dendritic cell vaccination, are conceptually attractive but remain early in development. Intrathecal chemotherapy and radiotherapy retain selected roles for CSF-directed treatment, symptom palliation, CSF flow restoration, and, in carefully selected good-risk patients, comprehensive coverage with proton CSI.

The literature is limited by retrospective designs, heterogeneous populations, variable LM diagnostic criteria, and small molecular subgroups, especially ROS1, BRAF V600E, NTRK, and RET alterations. Future studies should prospectively include dedicated LM cohorts, use LM-specific endpoints, validate CSF liquid biopsy for diagnosis and resistance profiling, and harmonize treatment and response assessment. Given the rarity of oncogene-driven LM, international multicenter collaboration is essential.

## Figures and Tables

**Table 1:** CNS and CSF accumulation of targeted therapies in preclinical and human data.

Drug	K_p,uu,brain_	K_p,uu,CSF_
EGFR		
Gefitinib	0.0021 ^a^	0.088 ^a^
Erlotinib	0.11 ^a^	0.29 ^a^
Afatinib	0.0066 ^a^	0.27 ^a^
Osimertinib	0.21–0.39 ^b^	0.29 ^a^
Lazertinib	0.087 ^a^	
ALK		
Crizotinib		0.0026 ^c^
Ceritinib	0.15 ^a^	
Alectinib	0.63–0.94 ^a^	
Brigatinib		
Lorlatinib		0.75 ^c^
ROS1		
Entrectinib	0.43 ^b^	
Lorlatinib		0.75 ^c^
MET		
Capmatinib		0.34 ^a^
Tepotinib	0.25 ^a^	

^a^ As measured in rats. ^b^ As measured in mice. ^c^ As measured in humans.

**Table 2:** Selected studies on EGFR TKIs in patients with LM from EGFR mutated NSCLC.

Publication	Type of Study	No. of Patients	Patient Characteristics	Prior Therapy	Treatment Regimen	Response to Therapy
Yang et al. BLOOM study [42]	Phase I clinical trial (2020)	41	EGFR-positive NSCLC with LM: 21 unselected, 20 with T790M mutation, 29 with brain metastasis	All had prior EGFR therapy 35 received prior chemotherapy	Osimertinib 160 mg daily	BICR according to RANO-LM criteria: LM ORR was 62%; median LM DoR was 15.2 months; mPFS by investigator was 8.6 months, mOS was 11.0 months.
Park et al. [43]	Phase II (2020) (LM cohort)	40	EGFR-positive NSCLC with LM: with and without brain metastasis	All patients had been treated with one or two lines of TKIs priorly, including standard dose osimertinib 80 mg daily	Osimertinib 160 mg daily	Intracranial DCR was 92.5%; CR, 12,5% and non-CR/non-PD, 80%. mPFS was 8.0 months, mOS was 13.3 months.
Ahn et al. [44]	Retrospective analysis (2020)	22	EGFR T790M- positive NSCLC with LM	All had prior EGFR therapy. 64% of patients had cytotoxic chemotherapy. 41% of patients had radiotherapy	Osimertinib 80 mg daily	BICR according to RANO-LM criteria: LM ORR was 55%, DCR was 91%. mPFS was 11.1 months, mOS was 18.8 months.
Saboundji et al. [45]	Retrospective analysis (2018)	20	EGFR-positive NSCLC with LM: 65% with T790M mutation	All had prior EGFR therapy.	Osimertinib 80 mg daily (*n* = 17), 160 mg daily (*n* = 2), 40 mg daily (*n* = 1)	Among the 11 radiologically assessable patients, 82% responded. mPFS was 17.2 months, mOS was 18 months.
Gtommres et al. [39]	Retrospective analysis (2011)	9	EGFR-positive NSCLC with CNS metastases (brain and/or LM): 1 with brain only, 8 with LM with or without brain metastasis	All had prior EGFR therapy.	Erlotinib 1500 mg once per week	CNS radiographic response using RECIST 1.1: CNS ORR was 67%. mOS was 12 months.
Kawamura et al. [40]	Retrospective analysis (2015)	12	EGFR-positive NSCLC with LM: 1 with T790M mutation in 7 patients	All had prior EGFR therapy.	200 mg on alternate days (*n* = 1), 300 mg on alternate days (*n* = 6), 300 mg every 3 days (*n* = 1), 450 mg every 3 days (*n* = 3), or 600 mg every 4 days (*n* = 1)	MRI responses: 3 (30%) of 10 evaluable patients, neurological symptoms improved in 6 (50%) of 12 patients. mOS was 6.2 months.

ORR—overall response rate; mPFS—median progression free survival; mOS—median overall survival.

**Table 3:** Ongoing Clinical Trials of ADCs.

Trial No.	ADC	Target	Disease	Phase/Enrollment	Primary Objective
NCT05865990 (TUXEDO-3)	Patritumab Deruxtecan	HER3	Solid tumor leptomeningeal disease	Ph 2/63	Intracranial objective response rate (ORR-IC) by RANO
NCT06250777 (ELPIS)	Trastuzumab Deruxtecan	HER2	Advanced or metastatic non-squamous NSCLC with HER2 mutation with asymptomatic brain metastasis	Ph 2/27	Median intracranial progression-free survival (icPFS) by RANO

## Data Availability

No new data were created or analyzed in this study.
